# The genetic architecture of youth anxiety: a study protocol

**DOI:** 10.1186/s12888-024-05583-9

**Published:** 2024-02-23

**Authors:** Laina McAusland, Christie L. Burton, Alexa Bagnell, Khrista Boylan, Taylor Hatchard, Patricia Lingley-Pottie, Abdullah Al Maruf, Patrick McGrath, Amanda S. Newton, Karen Rowa, Russell J. Schachar, S-M Shaheen, Sam Stewart, Paul D. Arnold, Jennifer Crosbie, Manuel Mattheisen, Noam Soreni, S. Evelyn Stewart, Sandra Meier

**Affiliations:** 1 The Mathison Centre for Mental Health Research & Education, Hotchkiss Brain Institute, Cumming School of Medicine, University of Calgary, Calgary, AB Canada; 2 Department of Psychiatry, University of Calgary, Calgary, AB Canada; 3 Department of Medical Genetics, University of Calgary, Calgary, AB Canada; 4 Neurosciences & Mental Health, Hospital for Sick Children, Toronto, ON Canada; 5 Department of Psychiatry, Dalhousie University, Halifax, NS Canada; 6 Department of Psychiatry and Behavioural Neurosciences, McMaster University, Hamilton, ON Canada; 7 Offord Center for Child Studies, Hamilton, ON Canada; 8 Child and Youth Mental Health Program, Hamilton Health Sciences, Hamilton, ON Canada; 9 Youth Wellness Center, St. Joseph’s Healthcare, Hamilton, ON Canada; 10 Department of Psychiatry, IWK Health Centre, Halifax, NS Canada; 11 College of Pharmacy, Rady Faculty of Health Sciences, University of Manitoba, Winnipeg, MB Canada; 12 Department of Pediatrics, University of Alberta, Edmonton, AB Canada; 13 Anxiety Treatment and Research Clinic, St. Joseph’s Healthcare Hamilton, Hamilton, ON Canada; 14 Department of Psychology, Neuroscience, and Behaviour, McMaster University, Hamilton, ON Canada; 15 Department of Psychiatry, University of Toronto, Toronto, ON Canada; 16 Department of Epidemiology and Community Health, Dalhousie University, Halifax, NS Canada; 17 Alberta Children’s Hospital Research Institute, University of Calgary, Calgary, AB Canada; 18 Department of Computer Science, Dalhousie University, Halifax, NS Canada; 19 Pediatric OCD Consultation Service, St. Joseph’s Healthcare Hamilton, Hamilton, ON Canada; 20 British Columbia Children’s Hospital Research Institute, Vancouver, BC Canada; 21 Department of Psychiatry, University of British Columbia, Vancouver, BC Canada

**Keywords:** Genetics, Anxiety disorders, Child & adolescent psychiatry

## Abstract

**Background:**

Anxiety disorders are the most common psychiatric problems among Canadian youth and typically have an onset in childhood or adolescence. They are characterized by high rates of relapse and chronicity, often resulting in substantial impairment across the lifespan. Genetic factors play an important role in the vulnerability toward anxiety disorders. However, genetic contribution to anxiety in youth is not well understood and can change across developmental stages. Large-scale genetic studies of youth are needed with detailed assessments of symptoms of anxiety disorders and their major comorbidities to inform early intervention or preventative strategies and suggest novel targets for therapeutics and personalization of care.

**Methods:**

The Genetic Architecture of Youth Anxiety (GAYA) study is a Pan-Canadian effort of clinical and genetic experts with specific recruitment sites in Calgary, Halifax, Hamilton, Toronto, and Vancouver. Youth aged 10–19 (*n* = 13,000) will be recruited from both clinical and community settings and will provide saliva samples, complete online questionnaires on demographics, symptoms of mental health concerns, and behavioural inhibition, and complete neurocognitive tasks. A subset of youth will be offered access to a self-managed Internet-based cognitive behavioral therapy resource. Analyses will focus on the identification of novel genetic risk loci for anxiety disorders in youth and assess how much of the genetic risk for anxiety disorders is unique or shared across the life span.

**Discussion:**

Results will substantially inform early intervention or preventative strategies and suggest novel targets for therapeutics and personalization of care. Given that the GAYA study will be the biggest genomic study of anxiety disorders in youth in Canada, this project will further foster collaborations nationally and across the world.

## Background

Anxiety disorders are currently the most prevalent class of psychiatric disorders worldwide, impacting an estimated 4.1% of 10-19-year-olds [1]. Canadian youth have an estimated six-month prevalence of 11 to 15% [2] with the most frequent diagnoses being separation anxiety disorder, specific phobias, social anxiety disorder, generalized anxiety disorder (GAD), panic disorder, and agoraphobia. Over the last three decades, a steep increase in the prevalence of anxiety disorders has been observed [3]. Considering the impact of the COVID-19 pandemic [4], this trend is likely to continue [5]. These disorders typically have an early onset in childhood/adolescence resulting in substantial impairment across the lifespan [6–9].

Anxiety disorders commonly co-occur; multiple correlations have been identified among different anxiety disorders [10], particularly between agoraphobia and social anxiety disorder (*r* = 0.68), panic disorder (*r* = 0.64), and specific phobia (*r* = 0.57), and specific phobia and social anxiety disorder (*r* = 0.50). High current and lifetime comorbidities are also observed with other psychiatric disorders, especially depression as over 50% of individuals with depressive disorders report a history of an anxiety disorder [11]. Substantial overlap has also been observed with post-traumatic stress disorder (PTSD), obsessive-compulsive disorder (OCD), substance use disorders, and attention deficit/hyperactivity disorder (ADHD) [10].

Both genetic and environmental factors play an important role in the intricate pathogenesis of anxiety disorders; in particular, genetic factors account for the moderate stability of anxiety disorders across the lifespan [12]. Current heritability estimates converge to rates around 35% for GAD and around 50% for social anxiety disorder, panic disorder, and agoraphobia [13]. The mode of inheritance is complex, with many genetic variants of small effect interacting with, or adding to other (environmental) risk factors [14, 15]. Importantly, heritability estimates of child and adult anxiety measures differ [16]. Longitudinal twin studies suggest that heritability is high in childhood but decreases over adolescence and into adulthood [12, 17, 18]. The genetic structure of anxiety disorders also seems to change across development. Anxiety subtypes in adults seem to fit a 2-factor model characterized by distress (GAD and depression) and fear (panic disorder and specific phobias) [19], but different structures have been found in youth with different genetic influences on anxiety and depression in childhood, common genetic vulnerability for anxiety and depression emerging in adolescence, and broadening associations in young adulthood [20].

The most well-researched source of genetic variation known to influence the risk of psychiatric disorders are common single nucleotide polymorphisms (SNPs). Genome-wide association study (GWAS), which enables the search for risk variants across the genome, is ideally suited to study common genetic risk factors for polygenic conditions such as anxiety disorders. GWAS for specific anxiety disorders and traits were historically severely underpowered [13]. To overcome sample size limitations, researchers started analyzing disorder subtypes together. By meta-analyzing the results of 7 GWAS on 5 clinically ascertained anxiety disorder subtypes (*n* = 17,310), the ANGST Consortium study [21] identified 2 genome-wide significant loci. A GWAS in the UK biobank on composite anxiety phenotypes using self-reported symptoms and diagnoses (*n* = 83,566) identified 5 genome-wide significant loci [22]. In addition, the largest anxiety GWAS to date was performed in 175,163 European and 24,448 African military veterans using a 2-item dimensional measure of GAD [23]. The study identified 6 significant loci for anxiety in European Americans and one in African Americans. But GWAS studies of anxiety phenotypes in youth have thus far been unsuccessful in identifying any genome-wide significant loci due to reasons such as low power and heterogeneity [24–26].

Genetic correlations can guide our understanding of the nature and patterns underlying complex traits and disorders. Large GWAS of anxiety disorders show strong positive genetic correlations with major depressive disorder (MDD) (rG = 0.78) [22, 23]. Accounting for comorbid MDD results in diminished but still significant SNP-based heritability for anxiety symptoms [23], indicating shared but also specific genetic effects of MDD and anxiety. Genetic correlations have additionally been observed between anxiety and other psychiatric disorders (e.g., schizophrenia, ADHD), sleep, and cardiometabolic traits and risk factors [22, 23]. GWAS of internalizing disorders in youth showed strong genetic correlations (rG > 0.7) with adult anxiety. However, the observed correlations with adult anxiety disorders were partial rather than complete, indicating that from a developmental perspective, childhood/adolescent internalizing symptoms are not genetically identical to adult anxiety or depression [25]. Given these differences, further clarification of specific genetic contributions to youth anxiety is needed.

Behavioural and cognitive traits, particularly behavioral inhibition (BI), inhibitory control and avoidance, are known to confer risk to later development of anxiety disorders. BI is a strong vulnerability marker of anxiety [27, 28] and is defined as an early childhood temperament characterized by shyness, fear, negative reactions to novelty, and avoidance of unfamiliar contexts or people [29–31]. Although BI is the best-known risk factor for anxiety disorders and associated with a 4–6 fold increased risk [32], only an estimated 40% of behaviourally inhibited children will develop anxiety disorders [33]. Research suggests that inhibitory control (the ability to inhibit responses to goal-irrelevant stimuli) plays a moderating role in the trajectory from childhood BI to adulthood anxiety disorders. For example, youth who inhibit their impulses best typically develop anxiety disorders in adulthood [34, 35], thus highlighting the need to assess both BI and inhibitory control in youth. Avoidance of stimuli or situations perceived as dangerous or threatening is a cardinal feature of anxiety disorders. This avoidance is self-reinforcing, shaping further retreat over time [36]. Avoidance is a primary intervention target. At its core, avoidant behavior is fueled by a desire to avoid danger, a feature that makes anxious youth vigilant for threat and prone to exaggerate their interpretations of it. Risk avoidance is well studied in anxious adults [37, 38] and, to a lesser extent, in youth with anxiety [39–41]. Among factors that drive avoidant behavior, the aversion to risky behaviours might be of particular relevance in the etiology of anxiety disorders. Measuring inhibitory control and risk tolerance in the context of anxiety could help elucidate cognitive mechanisms underlying youth anxiety.

The first-line treatment option for anxiety disorders in youth is cognitive behavioural therapy (CBT) [42]. CBT involves psychoeducation about anxiety, teaches youth skills for managing fears (e.g., relaxation, cognitive restructuring, problem solving), and helps youth to gradually face their fears while minimizing avoidance (i.e., exposure) [43]. The effectiveness of CBT (face-to-face or online) for youth anxiety has been demonstrated in several randomized control trials indicating large pre- to post- treatment effects and demonstrating superiority over control conditions [44, 45]. Valid second line treatment options are medication monotherapy, i.e., selective serotonin reuptake inhibitors [46], as well as the combination of CBT and medication [47, 48]. Nonetheless, one in three youth fail to respond to existing treatments [49], and few remain in remission [50]. Many youths also do not seek and/or receive treatment [2]. Given that genetic risk factors can affect the clinical response of patients [51], the emerging field of therapygenetics may be particularly important in predicting treatment outcomes. Unfortunately, to date, samples of youth undergoing CBT for anxiety disorders are difficult to recruit and retain, and these analyses have so far been underpowered [52–54].

## Methods

### Aims

In the current article we outline the design and methods of the GAYA study that aims to better understand the genetic underpinnings of anxiety disorders in Canadian youth. The GAYA study will help close the above-identified gaps in existing research in youth anxiety through a framework of integrated specific aims that will enhance our understanding of the specific genetic contributions to anxiety from childhood through adolescence, and implications for treatment. The specific aims and hypotheses are outlined in Table 1.

**Table 1:** Specific aims and hypotheses

	Specific Aim	Hypothesis
**1**	Identify genetic risk factors associated with clinical symptoms and vulnerability markers of youth anxiety.	Each clinical symptom and vulnerability marker will be associated with common genetic variations.
**2**	Identify genetic factors that are unique to anxiety in different age groups.	If the overlap in genetic risk factors is partial between different age groups, then the non-overlapping part will be driven by associations with common genetic variants.
**3**	Identify genetic factors that are shared and unique between anxiety and its common comorbidities.	If pleiotropy is driving co-occurrence of anxiety and common comorbidities, then these traits will have shared genetic risks.
**4**	Identify a prediction model of treatment response in youth with anxiety disorders.	Response to CBT in youth anxiety will be predicted using data from common genetic variation, clinical symptoms, and vulnerability markers.

### Participants

The study will make use of a population-based design enriched for youth with anxiety disorders as this sampling scheme has been shown to result in the highest power per included individual for quantitative and categorical traits with limited risk of biases [55–58]. The goal is to recruit 13,000 youth aged 10–19 of which 50% are expected to endorse symptoms of anxiety that indicate the presence of an anxiety disorder. This will be achieved by sampling from clinical settings as well as the general population. Youth will be recruited across Canada to the GAYA study with local sites in Calgary, Halifax, Hamilton, and Vancouver. The Toronto site will recruit from existing participants of Spit for Science [59, 60] who have agreed to be contacted about other research studies. To maximize generalizability among youth, GAYA assumes broad inclusion criteria and no exclusion criteria. Participants are eligible if they are (1) aged 10–19 years, (2) able to speak and understand English, and (3) have access to and are comfortable using a tablet or smartphone. These criteria will ensure participants can communicate with study staff and complete all study procedures in the language and format available.

### Procedure

#### Clinical recruitment

Youth will be recruited via clinics (e.g., Calgary’s Child and Adolescent Addiction Mental Health and Psychiatry Program and the Summit: Marian & Jim Sinneave Centre for Youth Resilience, the IWK Health Centre community, school mental health clinics, the Lynwood Charlton Centre, McMaster Children’s Hospital, and BC Children’s Hospital Mental Health) and family doctor/general practitioner practices by displaying study posters and leaflets in the waiting areas, conducting mail-outs, approaching patients in the clinics or over the phone, and contacting participants who have enrolled in research registries in which they indicate their willingness to be contacted regarding future studies focused on child and youth mental health. This clinical recruitment approach will ensure that youth with moderate to severe anxiety disorders are well represented in the GAYA study.

#### Online recruitment

Given that only 20% of youth with anxiety disorders receive care [2], the second approach will focus on online recruitment. Due to their symptomatology anxiety patients are often underrepresented in studies requiring face-to-face contact [61]. However, such patients can be successfully engaged through online outreaches via social media channels [61]. As youth spend a large amount of their time online [62], especially those with anxiety disorders [63], it is expected that online recruitment strategies can be successfully employed in this age group [61]. Specifically, multiple focused and intense social media campaigns will be launched across Canada to inform the public about the GAYA study. Social media content and visuals will include a range of infographics and short videos, each designed to be informative and provide basic details for the public about the aims of GAYA and how to join the project. The social media content and visuals will be co-designed by youth. All advertisements will include a study link for youth interested in learning more about the study to be directed to the study site. A wide range of charities, professional bodies, social media influencers, and youth with lived experience will be involved in the distribution of the recruitment materials.

#### Community-based recruitment

The GAYA study will make use of community-based designs leveraging collaborations with the Telus Spark Science Centre in Calgary, the Discovery Centre in Halifax, and the Ontario Science Centre in Toronto [59, 60].

#### Standard operating procedures

Each site is utilizing a standard operating procedure (SOP) to ensure that all data is collected in a consistent manner. These include SOPs for in-person saliva sample kit collection, saliva kit preparation and posting, data and privacy protections, and communication to participants. Established SOPs with messaging for mail, social media, and e-mail communication will be used across the sites, and will be reviewed by clinicians to ensure accessability and understandability.

#### Screening & consent

Potential participants will contact the researchers through the GAYA website or using contact information provided on recruitment materials and will be screened for eligibility. Eligible youth will complete the consent form and may opt-in to long-term storage of their DNA sample (except for Toronto-based participants who have already provided their genetic sample) and anonymized data sharing. For youth unable to consent for themselves based on the decision-making capacity requirements of the ethics board at each individual study site (e.g., young age), parental consent will be obtained with youth assenting to participate.

The consent form will ask the participant to indicate their willingness to participate in the GAYA study. For both the parents and youth, the consent forms will explain the purpose of the study, its risks and benefits, and the time commitment involved in participation.

### Measures

**Fig. 1:**
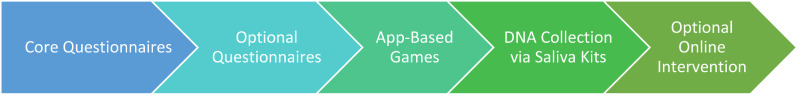
Stages of participant sign-up and involvement in the GAYA study

**Fig. 2:**
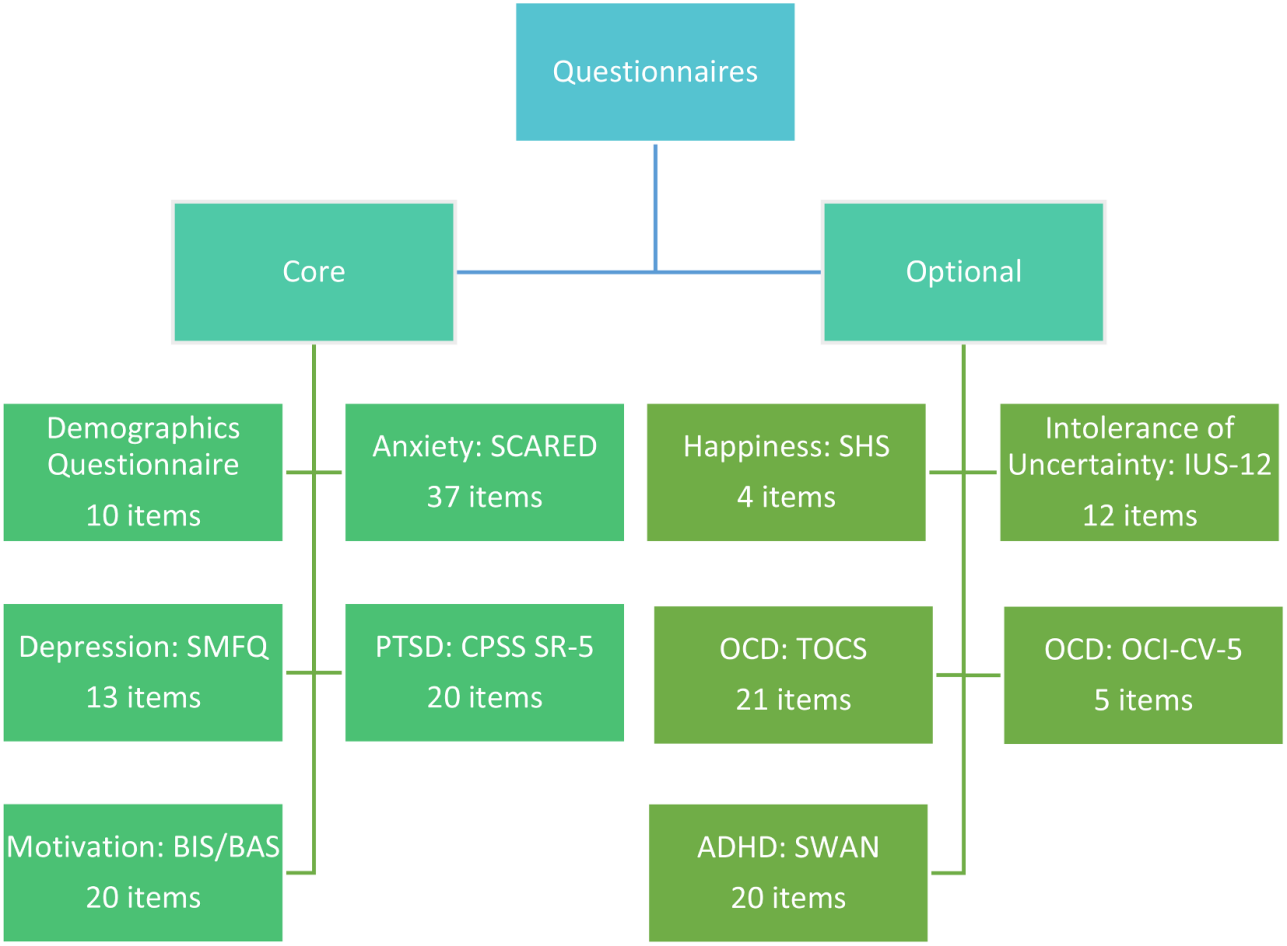
Questionnaires in the GAYA study

To limit the study burden for participants while simultaneously ensuring in-depth data collection, participants will be asked to complete five mandatory core self-report questionnaires and five optional self-report questionnaires. The core questionnaires will focus on anxiety, as well as common comorbidities and moderators known to be important in understanding the expression of anxiety. Youth will be able to complete all core questionnaires in 20–25 min. The optional questionnaires will further explore other relevant comorbidities and areas related to anxiety for participants who are willing to provide additional time. Youth will be able to complete all optional questionnaires in 10–15 min. The questionnaires will be completed online using REDCap [64, 65] electronic data capture tools hosted at the individual study sites, making use of an integrated ID management and pseudonymization framework. The flow of study tasks is displayed in Fig. 1. Details of core and optional questionnaires are displayed in Fig. 2.

#### Core questionnaires

The demographic questionnaire will be designed by the study team to gather information about the youth’s age, sex assigned at birth, gender, socioeconomic status, ethnicity, education, lifetime diagnoses of mental health disorders, and current/past mental health treatment history.

To assess anxiety symptoms, the study will use the Screen for Child Anxiety Related Emotional Disorders (SCARED [66]), validated for youth ages 9–18, which assesses symptoms of panic, GAD, separation anxiety, and social anxiety disorder, over the past 3 months. Each of the 41 items will be rated on a 3-point scale ranging from 0 to 2 (0 = hardly ever true, 1 = sometimes true, 2 = often true). The SCARED has high sensitivity (82%) and moderate specificity (52%), and acceptable-to-excellent internal consistency (α = 0.89–0.94), test-retest reliability (ICCs 0.59–0.86), reasonable convergent validity with anxiety measures, and discriminant validity [66–69].

Depression symptoms will be measured using the Short Mood and Feeling Questionnaire (SMFQ), a short version of the MFQ [70] validated for youth ages 6 and up, which was developed to briefly evaluate depressive symptoms in youth over the past 2 weeks [71]. The SMFQ consists of 13 items. Each item will be rated on a 3-point scale ranging from 0 to 2 (0 = not true, 1 = sometimes true, 2 = true). The SMFQ has high specificity (83%) and moderate sensitivity (71%), and acceptable-to-excellent internal consistency (α = 0.83–0.92), test-retest reliability (ICCs 0.61–0.80), and reasonable convergent validity with other depression measures [72–76].

Post-traumatic stress disorder (PTSD) symptoms will be measured using the CPSS-SR-5, validated for youth ages 8–18. It is a modified version of Child PTSD Symptom Scale self-report (CPSS-SR) [77] for DSM-5. The 20 PTSD symptom items will be rated over the past month on a 5-point scale of frequency and severity from 0 (not at all) to 4 (6 or more times a week/severe). The CPSS-SR-5 has excellent internal consistency for total symptom severity (α = 0.92), good test-retest reliability (*r* = 0.80), and high sensitivity (93%) and specificity (82%) for a probable diagnosis of PTSD. The CPSS-SR-5 also demonstrates convergent validity with the CPSS-I-5 interview(*r* = 0.90) [77–79].

Behavioural inhibition will be evaluated using the Behavioural Inhibition and Behavioural Activation System (BIS/BAS) scales, validated for ages 8 and up, which were designed to measure individual differences in motivational systems that impact behavior and affect [80]. The BIS is understood to be characterized by inhibitory responses in circumstances where cues signalling aversive consequences are present whereas the BAS system is characterized by responding to cues of reward, escape, and avoidance. The 20 items will be completed using a 4-point scale from 1 (disagree strongly) to 4 (agree strongly). Factor analysis revealed a single 7-item scale designed to assess BIS features, and 3 scales, Reward Responsivity (5 items), Drive (4 items), and Fun Seeking (4 items) that assess different aspects of BAS functioning [81]. The BIS/BAS scales have good internal consistency (α = 0.62–0.88), test-retest reliability (*r* = 0.53-86), reasonable convergent validity with anxiety/neuroticism measures, and discriminant validity [81–83].

#### Optional questionnaires

Happiness will be measured using the Subjective Happiness Scale (SHS) [84], a self-report measure of subjective happiness validated in ages 14–94. The scale includes 4 items rated on a 7-point Likert scale ranging from 1 to 7. Previous research has demonstrated that the SHS has good internal consistency (Cronbach’s α > 0.78) and test-retest reliability (Cronbach’s α > 0.54) [85].

Fear and anxiety of the unknown will be evaluated using the Intolerance of Uncertainty Scale (12-item) (IUS-12), a self-report measure of intolerance of uncertainty in children and adolescents validated for ages 8 and up. The scale includes 12 items rated on a 5-point Likert scale ranging from 1 (i.e., Not at all characteristics of me) to 5 (i.e., Entirely characteristic of me). The IUS-12 has demonstrated good psychometric properties in a variety of samples (e.g., Cronbach’s α = 0.93) [86].

Obsessive compulsive disorder (OCD) symptoms will be assessed using the Toronto Obsessive-Compulsive Scale (TOCS) which measures OCD traits from the past 6 months and is validated for youth ages 6–21. The 21 items are scored on a 7-point scale ranging from − 3 (far less often than average) to + 3 (far more often than average). The TOCS has excellent internal consistency (α = 0.94), and convergent and discriminant validity [87] with other OCD measures, as well as excellent sensitivity (77–92%) and specificity (92–98%) [88]. We also measure OCD using the 5-Item Obsessive-Compulsive Inventory– Child Version Scale (OCI-CV-5). It is a self-report measure of obsessive-compulsive disorder (OCD) symptomatology in children and adolescents, validated for ages 7–17. The scale includes 5 items rated on a 3-point Likert scale ranging from 0 (i.e., never) to 2 (i.e., always). In previous research, the OCI-CV has demonstrated good internal reliability (Cronbach’s α > 0.85) and internal consistency (Cronbach’s α = 0.91) [89].

The Strengths and Weaknesses of ADHD symptoms and Normal Behaviour Rating Scale (SWAN), validated for youth ages 6–17, will be used to measure attention, hyperactivity, and impulsivity traits [90]. This scale includes 30 items scored from − 3 to + 3 (below average to above average), where 0 (zero) is normal and based upon the population average. The SWAN has high sensitivity (86%) and specificity (94%), internal consistency (α = 0.88-0.98) [91, 92], test-retest reliability (ICCs 0.84–0.90, Pearson’s *r* = 0.72–0.90) [93, 94], reasonable convergent validity with ADHD measures [93, 94], and discriminant validity [89]. The SWAN and its subscales were found to be heritable (24–40%) and associated with ADHD polygenic risk [90].

#### Neurocognitive assessment app

Given the relationship between anxiety and inhibitory control and risk avoidance, youth will complete two neurocognitive tasks to measure these domains. One of the best-known paradigms to test inhibitory control is the Eriksen flanker task [95]. The flanker task requires learning novel associations between certain stimulus properties and responses to perform the task. Irrelevant stimuli must be inhibited while responding to the relevant target stimuli. The flanker task has been validated across varying development stages [96–98] and is frequently used in studies involving youth patients with anxiety disorders [99–101]. We created a youth-friendly version of this classical paradigm [102]. Youth will be presented with five fish and instructed to identify the swimming direction of the middle fish (relevant target stimuli) as quickly and accurately as possible while disregarding other fish (irrelevant stimuli) using a button in the app. The task estimates inhibitory control based on reaction times and accuracy.

The second task is the youth friendly Balloon Analogue Risk Task (BART) [103], a widely used neurocognitive paradigm to study risk-taking behaviour [104, 105]. The BART has been demonstrated to validly measure risk-taking in children, adolescents and adults; [105–108] it is also sensitive to development-related changes in risk-taking [109]. Presented with a balloon, youth can repeatedly pump up the balloon to increase their earnings or stop pumping and collect their accumulated earnings. The more pumps the participant makes increases the likelihood that the balloon will pop, resulting in a loss of all earnings. The average number of pumps across all trials will be recorded with increased pumps reflecting increased risk-taking proclivity [110] and lower numbers indicating increased risk avoidance.

As youth with anxiety disorders are likely to be affected by the presence of an unknown examiner [111], and to increase accessibility, we developed the GAYA app for administering the neurocognitive tasks. Co- designed with youth, the GAYA app allows youth participants to complete the tasks remotely in an environment of their choosing. The app can be installed on smartphones and tablets with iOS or Android operating systems. Participating youth will be provided with a download link for the app and their personal login credentials by the study team.

#### Saliva samples

Saliva sample collection will follow established protocols. Saliva-derived DNA has been shown to perform nearly as well as blood DNA [112, 113] and is routinely used in large-scale genetic studies in youth [60]. Youth will be invited to provide saliva samples at the nearest individual recruitment sites (Calgary, Halifax, Hamilton, or Vancouver) with an OG-600 Oragene saliva DNA sample kit or choose to have an OCR-100 ORAcollect DNA sample kit mailed to their home with a pre-paid return envelope. De-identified research IDs will link data and saliva samples. The linked IDs will be logged in REDCap to document and track the handling of biologic samples. Participants will be instructed on how to provide a sample by a trained research staff member via live video or a pre-recorded video and receive written instructions. Toronto participants will have already provided their saliva samples at the time of participating in Spit for Science using OG-600 Oragene saliva DNA sample kits.

### Optional intervention

#### Intervention procedures

Youth enrolled in GAYA who are ages 13–19, not currently in mental health treatment, and do not endorse psychosis screening questions will be offered the opportunity to participate in a self-managed Internet-based CBT (iCBT) program, Breathe (Being Real, Easing Anxiety: Tools Helping Electronically) [114], at all recruitment sites. These youth will be linked to the Strongest Families Institute (SFI, http://strongestfamilies.com/), where all referred youth will receive the Breathe program that is part of SFI’s validated eplatform IRIS (Intelligent Research and Intervention Software).

Through weekly self-managed check-ins, during which youth assess and rate their social-emotional functioning over the past week, a detailed monitoring of anxiety symptoms over the course of the Breathe program will be enabled. Youth will also be asked to rate their anxiety symptoms based on the SCARED pre-, post-treatment, and at a 3 months follow-up. Intervention response will be defined as the changes in SCARED scores from pre- to post-treatment for the 3 months follow-up.

#### Breathe intervention description

Breathe is a self-mediated 6-module standardized iCBT program that involves: (a) multimedia-based education about anxiety problems and approaches to overcoming anxiety (e.g., reviewing why exposure exercises are important); (b) self-assessment activities to determine level of intervention and safety needs; (c) activities that teach users about anxiety sensitivity and how to develop realistic thinking about anxiety-producing situations; (d) activities for practicing coping and relaxation skills; (e) development of a hierarchy of feared situations and steps for gradual and repeated exposure to feared situations (using imagery/in vivo activities); (f) contingency management (examining the function of anxiety from a reinforcement perspective) and modelling (viewing videos of others confronting feared situations); and (g) skills for maintenance and relapse prevention. Animations, embedded videos, timed prompts, and on-screen pop-ups are used in each module to provide an interactive and multimodal experience. In one of the largest effectiveness trials of iCBT in adolescents conducted to date, that used SFI’s IRIS eplatform, 563 adolescents aged 13–19 were randomly assigned to 6 weeks of Breathe (*n* = 280) or to visit a static (no elements of interactivity or personalization) website which provided resources for anxiety (*n* = 283) [115]. In the trial, adolescents who participated in Breathe had a greater improvement in symptoms 3 months after program use (*p* = 0.04) [115]. Although this Breathe study was complimented by one telephone coach support session to those who wanted the additional support, the current study will only be self-mediated.

### Collection of biomaterials, DNA extraction and genotyping process

DNA extraction from saliva is performed at each individual site following best practice and according to the manufacturers’ protocols. Subsequent genotyping will be performed in 3 batches. All individuals will be genotyped (using DNA from the saliva samples) with Illumina’s Global Screening Array v3.0 (GSA). The GSA is a cost-effective genotyping array that is routinely used for population-scale genetic studies around the globe. For genotyping calling Illumina’s GenomeStudio will be used. Spit for Science genotyping will be done locally using the same Illumina array and their genetic data is sent to the Halifax site once the participant has completed the questionnaires and/or app portion of the study.

### Quality control (QC) and imputation of GWAS data

The Ricopili pipeline will be used to perform QC of the genetic data within and across ancestral stratified subgroups (based on demographic information) [116]. Ricopili has been extensively used by international consortia for their large-scale GWAS and makes use of well-established analytic software during its processing steps (e.g., PLINK [116]). Analyses will look for any (hidden) *relatedness* or sample duplicates as part of our QC and flag individuals that are related (pi_hat > 0.2) for downstream analyses. Population substructure will be (re-) examined by principal components (PC) estimation and support vector machines will be run in joined PC analyses with a reference sample of known ancestral background (TopMed) to annotate the sample with population substructure information and compare this information with the demographic information collected during the online assessment. For *imputation*, a pre-phasing/imputation stepwise approach as implemented in Ricopili [117] will be used with and across population subgroups identified in our sample. This will include the evaluation of a potential increase in power through usage of imputation approaches that use local ancestry to enable inclusion of admixed individuals in the GWAS [118]. *ChrX* imputation will be conducted separately by sex for subjects passing an additional QC designed for these purposes [119]. For downstream analyses SNPs that have an INFO > 0.8 and a MAF > 0.01 will be considered.

### Data analysis strategy

Appropriate covariates (e.g., age, sex, gender, recruitment procedure/site) will be included for analyses in each trait. Where necessary and appropriate analyses will control for current/past treatment history through established protocols. For all GWAS, the impact of population substructure on the genome-wide test statistics using λGC [120] and Linkage Disequilibrium score regression (LDSC) analyses [121] will be evaluated. There are clear sex differences described in the epidemiology of anxiety. Anxiety disorders and symptoms occur more often in women, and the odds of developing an anxiety disorder is 1.7 times greater for women than men [122]. The analyses will therefore be stratified by sex and gender to explore shared and unique genetic contributions.

Data analysis will be aligned with each of the specific aims. For all results identified in aims 1–4, using MAGMA [123] and LDSC [124] tissue and single cell enrichment analyses will be conducted compiling publicly available single-cell RNA-sequencing data from five studies of the human and mouse brain [125–128]. Similarly, transcriptome-wide association studies will be conducted using FUSION [129] and expression quantitative trait locus data from the PsychENCODE Consortium (1,321 brain samples) [130]. In addition, analyses will include EpiXcan [131], an elastic net-based method, which weighs SNPs based on epigenetic annotation information [132].

#### Specific aim 1: identify genetic risk factors associated with clinical symptoms and vulnerability markers of youth anxiety

Following best practices in the field, additive model GWAS analyses will be conducted for common SNPs and each quantitative trait. To increase power, multivariate-based approaches will be employed that enable us to address the complex relationship of the anxiety phenotypes (SCARED subscales, BIS/BAS subscales, inhibitory control, and risk avoidance) amongst each other but also in relationship to the genetic data. As such the GW-SEM software package [133, 134] will be used. Genome-wide results from GAYA study samples will be meta-analysed with other available samples [25, 61] using inverse-variance weighting with METAL [135] and accounting for population structure. Sensitivity analyses using structural equation modelling (SEM), via genomic SEM, will help to address potential heterogeneity. Established approaches (LDSC [120] and GCTA [136]) will be used to study liability-scale heritability of clinical symptoms and vulnerability markers of youth anxiety. Partitioned heritability across minor allele frequency bins and functional annotations (e.g., cell-types) using the same software packages and publicly available data (e.g., from the PsychENCODE consortium [137]) will provide further insights into the genetic relationship of different clinical symptoms and vulnerability.

#### Specific Aim 2: identify genetic factors that are unique to anxiety in different age groups

Multi-trait conditional and joint analysis [138] to adjust GWAS summary statistics from the GWAS in youth for the genetic effects in adult anxiety to identify putative age group-specific SNP associations. It is noteworthy that previous analyses in closely related traits of similar sample size (e.g., ADHD) were able to identify new genome-wide significant hits specific to the traits under analysis [138]. Similarly, summary statistics from youth and adult anxiety GWASs will be analysed with ccGWAS [139], a tool designed to identify loci with different allele frequencies among different trait groups. Using ccGWAS genetic loci will be identified that are specific in their association to the individual age groups.

Using established protocols (SBayesR [140]/LDpred2 [141]/PRSice2 [142]) genomic risk profile scores (GRPS) will be generated in the GAYA study sample trained on discovery datasets from different age groups (i.e., pairwise between the youth and adult GWAS) to assess the variance explained through genetic liability for anxiety disorders in one age group for the other. Finally, where appropriate, CLiP [143] will be used to study heterogeneity in GRPS for the GAYA study sample.

#### Specific aim 3: identify genetic factors that are shared and unique between anxiety and its common comorbidities

Via LDSC [120] patterns of genetic correlation, common comorbidities (e.g., MDD, ADHD) with youth anxiety will be analyzed. Genomic SEM will be run including the youth anxiety GWAS along with the newest adult anxiety GWAS [21–23] to investigate the multivariate genetic architecture across youth anxiety and its comorbidities. In this multivariate GWAS, it will be possible to identify loci that confer risk to multiple disorders (i.e., that are shared across disorders).Two-sample Mendelian Randomization (MR) analyses will be conducted using the inverse-variance-weighted (IVW) MR method to investigate associations between the genetic liability for youth anxiety and adult-onset mental disorders, while further ensuring the robustness of our IVW estimates through MREgger and the MR robust adjusted profile score approach [144, 145].

#### Specific aim 4: identify a prediction model of treatment response in youth with anxiety disorders

GWAS data for general susceptibility for major psychiatric illnesses (such as adult and youth anxiety, MDD, ADHD, and others), and antidepressant treatment response [146] will be used to train GRPS in the GAYA study sample. For each of these GRPS the amount of variation explained in the clinical response of youth receiving Breathe will be assessed. It will also be evaluated how much of this variation can be explained by clinical symptoms/vulnerability markers and subsequently whether the combination of these measures (i.e., GRPS plus clinical symptoms/vulnerability markers) can increase our ability to predict clinical response in youth with anxiety disorders. Further, explorative GWAS will be conducted in two datasets: (a) 3,000 youth recruited to receive CBT and (b) around 10,000 individuals (all age groups, including the 3,000 youth) by combining the GAYA study sample with samples available via collaborations [52, 61].

### Youth council

A national youth council, including members of established youth councils at study sites, will consult through all phases of the design and management of the study. Meetings will be conducted virtually to allow for geographic diversity and youth council recruitment will prioritize representation of diverse demographics. Youth will advise on several aspects of GAYA, including recruitment strategies (i.e., flyers and posters); contact management and retention tools; study measures and instruments; assessment instrument package; and the assessment package’s length and readability. Additionally, as part of the knowledge translation plan, youth will be included in the interpretation of findings and their presentation through various knowledge translation activities, such as presentations, publications, short videos, infographics, and webinars co-led by youth.

## Discussion

While anxiety disorders have become more common in youth over the years and exacerbated by the pandemic, efforts aiming to explore the genetic underpinnings of anxiety disorders are limited. Twin studies strongly suggest that genetic susceptibility plays a role in the development of anxiety disorders and that this role is age-dependent [12, 17, 18] Thus, the GAYA study has the potential to fill an important gap in our current knowledge. Study results will significantly contribute to a better understanding of the developmental trajectory of anxiety disorders and its common comorbidities, increasing knowledge in relation to the high rates of co-occurrence observed across psychiatric disorders [22, 23]. As analyses of youth undergoing CBT for anxiety disorders have been previously underpowered [52–54], the GAYA study’s sample size will further inform prediction models of treatment response. Finally, study results are expected to inform early intervention or preventative strategies and suggest novel targets for therapeutics and personalization of care.

## Data Availability

No datasets were generated or analysed during the current study.

## Abbreviations

ADHD: Attention deficit/hyperactivity disorder
BART: Balloon Analogue Risk Task
BI: Behavioral inhibition
BAS: Behvaioural Activation System
BIS: Behavioural Inhibition System
Breathe: Being Real, Easing Anxiety: Tools Helping Electronically
CBT: Cognitive behavioral therapy
CPSS-SR: Child PTSD Symptom Scale self–report
GAD: Generalized anxiety disorder
GAYA: Genetic Architecture of Youth Anxiety
GRPS: Genomic risk profile scores
GSA: Global Screening Array
GWAS: Genome wide association study
iCBT: Internet–based cognitive behavioral therapy
IRIS: Intelligent Research and Intervention Software
IUS-12: Intolerance of Uncertainty Scale
IVW: Inverse–variance–weighted
LDSC: Linkage Disequilibrium score regression
MDD: Major depressive disorder
MR: Mendelian Randomization
OCD: Obsessive–compulsive disorder
OCI-CV: Obsessive–Compulsive Inventory–Child Version Scale
PC: Principal components
PTSD: Post–traumatic stress disorder
QC: Quality control
SCARED: Screen for Child Anxiety Related Emotional Disorders
SEM: Structural equation modelling
SFI: Strongest Families Institute
SHS: Subjective Happiness Scale
SMFQ: Short Mood and Feeling Questionnaire
SNP: Single nucleotide polymorphism
SOP: Standard operating procedure
SWAN: The Strengths and Weaknesses of ADHD symptoms and Normal Behaviour Rating Scale
TOCS: Toronto Obsessive–Compulsive Scale
